# Patterns of transcriptomic aging in the hippocampus of rhesus macaques highlight midlife transitions

**DOI:** 10.1007/s11357-025-01834-z

**Published:** 2025-08-18

**Authors:** Tanner J. Anderson, Marina M. Watowich, Kenneth L. Chiou, Elisabeth A. Goldman, Sam Peterson, Jordan A. Anderson, Noah Snyder-Mackler, Lucia Carbone, Steven G. Kohama, Kirstin N. Sterner

**Affiliations:** 1 Department of Anthropology, University of Oregon, Eugene, OR USA; 2 Department of Biological Sciences, Vanderbilt University, Nashville, TN USA; 3 Department of Biology, University of Washington, Seattle, WA USA; 4 Center for Evolution and Medicine, Arizona State University, Tempe, AZ USA; 5 School of Life Sciences, Arizona State University, Tempe, AZ USA; 6 Department of Biology, University of Alabama at Birmingham, Birmingham, AL USA; 7 Cancer Early Detection Advanced Research Center, Knight Cancer Institute, Oregon Health & Science University, Portland, OR USA; 8 Division of Neuroscience, Oregon National Primate Research Center, Beaverton, OR USA; 9 Institute of Ecology and Evolution, University of Oregon, Eugene, OR USA; 10 School of Human Evolution and Social Change, Arizona State University, Tempe, AZ USA; 11 Neurodegenerative Disease Research Center, Arizona State University, Tempe, AZ USA; 12 Department of Medicine, KCVI, Oregon Health & Science University, Portland, OR USA; 13 Department of Molecular and Medical Genetics, Oregon Health & Science University, Portland, OR USA; 14 Division of Genetics, Oregon National Primate Research Center, Beaverton, OR USA

**Keywords:** Aging, Hippocampus, Rhesus macaques

## Abstract

Patterns of brain aging are generally conserved among primates; however, there is marked variation in the observed rate among individuals, species, and brain regions. The hippocampus is a region particularly susceptible to the aging process. To better understand how the hippocampus changes over the lifespan, we measured gene expression in 96 banked hippocampus samples from adult male and female rhesus macaques aged 3–35 years old. Importantly, our dataset included representation across adulthood allowing us to characterize age-related patterns in gene expression during midlife, a period often underrepresented in studies of aging. We used autoregressive integrated moving average models to examine age-associated changes in gene expression to identify 2679 differentially expressed genes (FDR < 0.05) that fit four broad patterns of expression: linearly upregulated or downregulated across age, and two clusters with nonlinear patterns. Importantly, the nonlinear clusters highlight transitions in expression trajectories centered around ~ 10 years of age (~ 30 years of age in humans) indicating an important period that may have a critical impact on hippocampal aging. Changes in gene expression variance across age found that genes in individuals > 20 years of age (> 50 years of age in humans) have greater variance in expression than individuals aged 10–20 years (FDR < 0.05). Collectively, our results highlight molecular changes occurring during midlife which may shape brain aging in longer lived primates and may offer insight into increased susceptibility to neurodegenerative disease in humans.

**Supplementary Information:**

The online version contains supplementary material available at 10.1007/s11357-025-01834-z.

## Introduction

Aging is a complex, multifaceted process which encompasses numerous molecular and cellular changes that take place over an organism’s lifespan [1]. These changes manifest at various levels, from organismal to tissue-specific, and their pace and severity can differ significantly across species. For example, some organisms exhibit negligible senescence, maintaining function and avoiding age-associated decline for much of their lives [2–5]. At the tissue level, aging affects organs differently, with some tissues undergoing rapid degeneration while others maintain relative stability. In humans, brain aging is particularly consequential because it is often associated with cognitive decline driven by dysfunction in cortical and subcortical regions, although there is clear variation in the pace of brain aging between individuals. This variation is influenced by genetic, environmental, and lifestyle factors, underscoring the complexity of the aging process [6, 7].

While considerable research has been dedicated to uncovering the molecular mechanisms that shape brain aging [8], much of our current understanding comes from studies that contrast young and old individuals e.g., [9, 10]. While these studies are invaluable, they have a limited ability to characterize nonlinear changes occurring over the lifespan and may thus miss critical midlife changes that shape the trajectory of aging later in life. Indeed, a growing body of research suggests that midlife is an important period for brain health outcomes in humans (e.g., cognitive decline & neurodegenerative disease) [8, 11–16]. For example, the association between vascular risk factors (e.g., elevated blood pressure & impaired glucose regulation) and development of Alzheimer’s disease in humans shows a stronger relationship when measured at mid-life compared to when measured at late-life suggesting a need to focus interventions at this time point [12].

The hippocampus is heavily involved in learning and memory; as such, it has a central role in functional aging and represents an important comparison for primate brain aging research [17, 18]. Normal hippocampal aging is characterized by neurobiological alterations including increased oxidative stress and neuroinflammation, altered gene expression, reduced neurogenesis, and reduced synaptic plasticity [18]. Indeed, the hippocampus is distinguished by its enhanced neuroplasticity compared to other brain regions, which makes it more susceptible to age-related damage [17]. The hippocampus is also the first brain region to suffer blood-brain barrier breakdown over the course of aging [19]. Despite its vulnerability to age-related changes, there is considerable interindividual variability in the timing and extent of decline, with the origins of this variability yet to be fully understood [18]. Elucidating the molecular mechanisms underlying age-related changes in the hippocampus is necessary to understand drivers of cognitive decline and neurodegeneration.

Research in humans faces limitations due to the difficulty of studying neurobiological processes directly in the brain over time, particularly at the molecular level. Rhesus macaques (*Macaca mulatta*) are nonhuman primates with similar life history and conserved patterns of brain aging to humans and provide a robust translational model for understanding age-related changes in the brain [20–26]. The rhesus macaque model allows for more controlled studies that better account for factors like diet, environment, and veterinary care to uncover mechanisms that may otherwise be inaccessible in human research. Using biobanked samples from a cross-section of rhesus macaques, we characterized patterns of gene expression in the rhesus hippocampus across the lifespan, which allowed us to identify midlife transitions in molecular brain aging that may relate to later brain aging.

## Methods

### Samples

We leveraged the tissue archive of the NIH/NIA-funded Aging Primate Resource at the Oregon National Primate Research Center (ONPRC) and generated RNAseq data from 96 banked hippocampus samples from rhesus macaques of different ages (3–35 years; females = 59 and males = 37) (Table S1 and Fig. S1). All animals included in this study were derived from the tissue distribution program at the ONPRC, which followed the recommendations of the 2013 Edition of the American Veterinary Medical Association Guidelines for the Euthanasia of Animals. Tissues were collected opportunistically from veterinary culls. Brains were flushed with saline, and the right hippocampus was isolated out of the medial temporal lobe, divided into thirds in the coronal plane, frozen in liquid nitrogen, and archived at − 80 °C.

Samples used for this study were collected and banked over the course of 24 years (from 1996 to 2020). For all 96 samples, the main body of the hippocampus was pulverized in its entirety (i.e., all regions of the hippocampus: CA regions, dentate etc., are represented in the sample). Importantly, all samples included in this study showed no evidence of CNS lesions nor aberrant behaviors suggestive of brain disorders.

### RNA extraction, library preparation and sequencing

A combined DNA/RNA extraction protocol (Qiagen DNA/RNA Allprep kit) was used to isolate genomic DNA and total RNA from these samples by the ONPRC Primate Genetics Core. Extractions were done in groups of 24 over the course of 1 day with sample order randomized to ensure not all ages/sexes were grouped together. RNAseq libraries were generated with the TruSeq Stranded Poly(A) + protocol. Quality control was performed on all libraries by Tapestation and qPCR. Libraries were split across three lanes of a NovaSeq XP 200 Cycle S4 Flow Cell and sequenced with 100-bp paired-end reads at the OHSU Massively Parallel Sequencing Shared Resource core. Samples had a relatively consistent yield with an average of approximately 100 million reads per sample. All raw sequence data utilized in this analysis are publicly available (Accession: PRJNA1164722).

### Data preprocessing

Data quality control checks of the RNAseq data were performed using FastQC [27]. Adapter and poor-quality base trimming was performed using Trimmomatic [28] in paired-end mode followed by an additional round of FastQC. Reads were then mapped to the rhesus macaque reference genome Mmul_10 (Accession: GCA_003339765.3) using *STAR* [29] two-pass mode (average mapping rate =  ~ 79%). We then used the featureCounts program [30] for quantification of raw counts of mRNA regions and the generation of a counts matrix. Data preprocessing was done using the University of Oregon’s high-performance computing cluster, Talapas. All subsequent data and statistical analyses were performed in R version 4.1.2. All code used in the analyses is available at (https://github.com/tannerndrsn4/Hippocampus-RNAseq-Nonlinear-Modeling).

### Read count normalization

Read count normalization and filtering was done using the R packages edgeR [31] and limma [32]. The initial filtering step retained genes with sufficient expression levels across samples to ensure robust analysis. Specifically, genes were included if they had a log-transformed Counts Per Million (CPM) value above 4 in at least 15 samples. The voom function from the limma package transformed the filtered data into log2-counts per million (logCPM) values. Filtering and normalization parameters resulted in a final data set of 9563 genes and 96 individuals for subsequent statistical analysis.

### Differential expression analysis

A growing body of work indicates that molecular aging may follow nonlinear trajectories [33–35]; however, the extent to which this occurs in the aging rhesus macaque hippocampus is unknown. To characterize both linear (e.g., upregulated or downregulated across age) and nonlinear (e.g., midlife shifts) patterns of gene expression across the lifespan, we used autoregressive integrated moving average models (ARIMAs)—following a similar approach outlined in [36] and [37]. Briefly, the best fitting ARIMA model, as identified by the lowest Akaike Information Criterion (AIC) value, is determined for each gene as a function of age. The approach then selects all models which exhibit gene expression trajectories which are significantly different (FDR < 0.05) from random fluctuations in expression across the lifespan [38]. These genes are thus deemed to exhibit significant nonzero trends in expression; henceforth, they are referred to as differentially expressed genes (DEGs). See Supplemental Methods for more detail on the modeling framework and implementation.

Prior to differential expression analysis, eight individuals were removed after discovering tissue contamination (see Supplemental Methods, Fig. S2 and Fig. S3). We then performed a single round of residualization in which both sex and technical effects were jointly regressed out from our final dataset of 88 individuals. Using a linear model, we estimated the impact of sex and technical effects on expression values, then extracted the residuals to create a dataset adjusted for sex and technical effects. This approach ensured that subsequent analyses focused on age-related variation in gene expression, independent of these technical and biological confounders (see Supplemental Methods and Fig. S4 for more detail). Differential expression analysis was performed using ARIMA models to identify DEGs and hierarchical clustering analysis was used to group DEGs into gene clusters which share similar expression trajectories. To estimate the age at which gene expression trajectories shifted in nonlinear clusters, we fit LOESS curves to average expression values and identified the inflection point as the age with the lowest or highest predicted expression along the smoothed curve.

### Transcriptional dysregulation during adulthood

We tested for greater variance in gene expression for older adults compared to younger adults by splitting our dataset into two age categories: 10–20 years (22 females and 13 males) and > 20 years (20 females and 9 males). We focused on mid-adulthood as gene expression mechanisms become more established post-development, making adulthood a more suitable period for distinguishing age-related dysregulation from developmental changes [39].

For all 9563 genes included in the differential expression analysis, we compared variance in expression between the age cohorts by performing *F*-tests on each gene while also regressing out the effects of sex and controlling for mean expression for each respective gene. To control for mean expression, we calculated mean expression for each gene in both age groups and applied a similarity threshold (0.5) to include only genes with comparable expression levels between groups (9159 genes). This approach allowed us to analyze all genes initially, while focusing variance testing specifically on genes with similar mean expression across age groups. We then performed multiple hypothesis testing correction on the output using the R package qvalue [40] retaining only genes which passed our significance threshold (FDR < 0.05).

In addition, we tested whether the mean variance in gene expression differed significantly between age groups by performing a paired *t*-test on log-transformed variances across all genes. Variance values for each gene were log-transformed to stabilize variance estimates, and the mean variance was calculated for each age group to summarize overall differences in variance magnitude.

### Gene ontology enrichment analysis

We tested for gene set enrichment on our DEGs as identified by the ARIMA approaches as well as genes determined to exhibit significant differences in variance of gene expression between age groups using an R interface for enrichR [41–44]. Fisher’s exact test was used to determine enrichment status followed by the Benjamini–Hochberg method for multiple hypothesis test correction. For each gene set enrichment analysis, we profiled significantly enriched (FDR < 0.05) GO Biological Processes as well as KEGG pathways.

### Transcription factor binding site enrichment analysis

We tested for transcription factor (TF) motif enrichment using ChIP-X Enrichment Analysis 3 (ChEA3) [45]. Briefly, ChIP-X Enrichment Analysis 3 (ChEA3) identifies transcription factors (TFs) likely to regulate a specified gene set by integrating multiple sources of evidence, including ChIP-seq datasets, co-expression networks, TF-target gene databases, and literature text mining. For each input gene set, ChEA3 ranks TFs based on their association across these sources, providing a comprehensive enrichment score that highlights the most relevant regulatory TFs. Genes for each expression cluster were submitted as independent queries to provide enrichment results for each respective expression cluster. For this analysis, we chose to focus on the top 10 enriched TFs for each respective query.

### Cell-type deconvolution analysis

As cell composition can affect gene expression profiles observed in bulk RNAseq data, we characterized cell-type proportions via cell-type deconvolution using CIBERSORTx [46]. This program requires the input of a signature matrix derived from rhesus macaque hippocampus single-cell RNAseq coupled with our bulk hippocampus RNAseq data to estimate cell-type proportions. To generate our signature matrix, we used data from the most recent rhesus macaque single-cell RNA-seq brain atlas, [47]. We retained all cells from the hippocampus and generated average gene expression profiles for each cell type—denoted at the “cell class” level in the [47] dataset—in the dataset using the AverageExpression function in Seurat [48–52]. We then used the resulting matrix as the signature matrix required by CIBERSORTx.

Deconvolution was performed on all 9563 genes included in our previous analyses and performed using recommended criteria for RNAseq data (e.g., disabled quantile normalization & 100 permutations for significance analysis). First, we quantified whether cell-type significantly differed across age in the hippocampus of rhesus macaques using linear regression on the estimated cell proportions as a function of age for each cell type. Secondly, we used the CIBERSORTx output to control for cell-type change across age in an additional ARIMA approach which aimed to characterize gene expression changes across age independent of cell-type or sex effects using a similar approach as described above.

## Results

### Genes differentially expressed across age fit four broad expression patterns

**Fig. 1:**
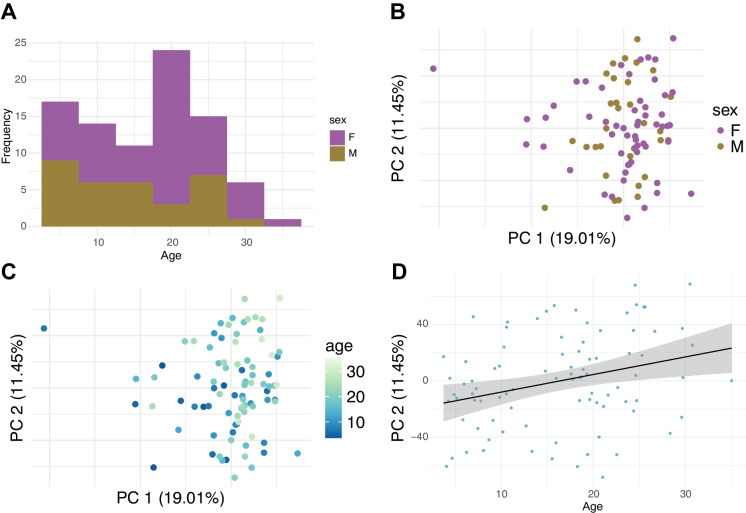
Sample distribution and PCA analysis for 88 rhesus macaque hippocampus samples. **A** Distribution of ages. Samples include representation across the lifespan and both males and females. Each bar represents an age range of 5 years. **B** PCA analysis of all 88 individuals included in differential expression analysis as denoted by sex, **C** and by age. **D** PC2 was found to be significantly associated with age (*β* = 0.6057, 95% CI [0.3603, 0.8511], *p* = 4.30e − 06)

Our preliminary data exploration highlighted a potential batch effect in the data along with the presence of outliers (see Supplemental Methods). Briefly, we removed eight individuals (Table S1) from the dataset (six individuals with evidence of choroid plexus tissue contamination and two extreme outliers) and corrected for technical effects, resulting in a final dataset of 88 individuals (55 female & 33 male) (Fig. 1A). We performed PCA analysis on this dataset (Fig. 1B and C) and found PC2 to be significantly associated with age (*β* = 0.6057, 95% CI [0.3603, 0.8511], *p* = 4.30e − 06) (Fig. 1D).

**Fig. 2:**
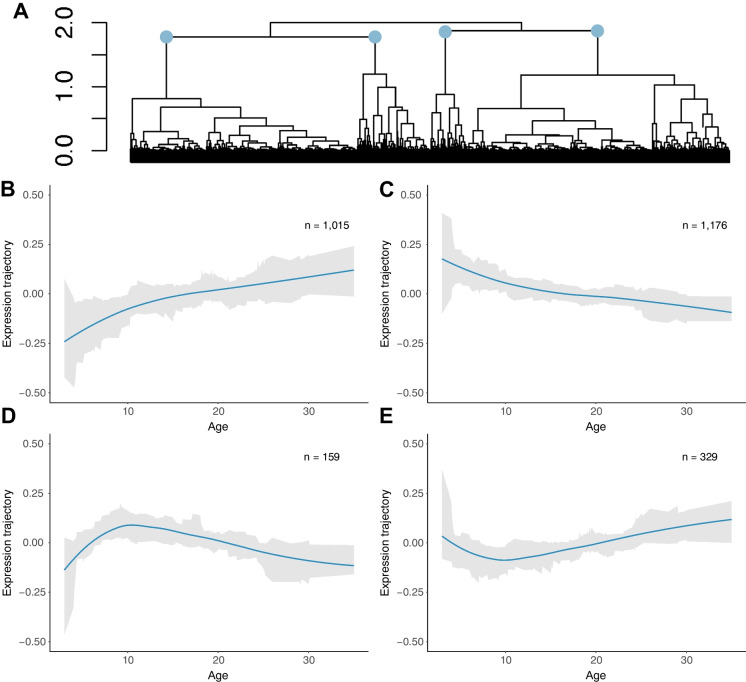
Hierarchical clustering and expression trajectories for DEGs that fit four broad expression patterns. **A** Cluster dendrogram of all significantly differentially expressed genes as identified by ARIMA modeling when controlling for sex. Clusters utilized in analysis are denoted by blue nodes. **B**–**E** Mean predicted expression counts for all genes in each cluster, as determined by ARIMA models. The blue line (LOESS-smoothed curve) represents the average trajectory, with a shaded ribbon highlighting ± 1 standard deviation around the mean. Number of genes in each cluster is denoted by *n*

We identified 2679 (28%) age-associated differentially expressed genes (DEGs) (FDR < 0.05) and identified four broad clusters in temporal gene expression using hierarchical clustering (Fig. 2A). Two clusters (cluster 1 [1015 genes; 37.9%] and cluster 2 [1176 genes; 43.9%]) demonstrated linear trends in gene expression (Fig. 2B and C respectively) while the other two (cluster 3 [159 genes; 5.9%] and cluster 4 [329 genes; 12.3%]) demonstrated nonlinear trends (Fig. 2D and E respectively).

**Fig. 3:**
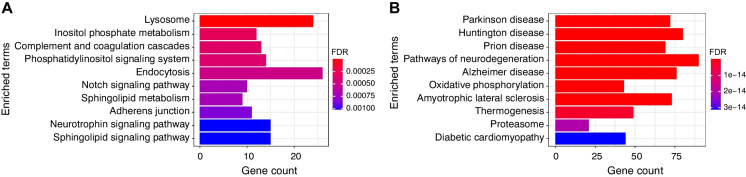
KEGG pathway enrichment analysis for genes exhibiting linear expression trajectories. **A** Top 10 enriched KEGG pathways after FDR correction (FDR < 0.05) for genes upregulated across age, and **B** genes downregulated across age using EnrichR

Cluster 1 contained 1015 genes (37.9% of DEGs) upregulated across age (Fig. 2B) and GO term analysis found enrichment for genes largely implicated in positive regulation of transcription and DNA-templated transcription (GO:0045893) and sphingolipid metabolic process (GO:0006665) (Table S2). Enriched KEGG pathways contained 15 significant pathways after FDR correction, including CNS-related terms (e.g., Notch signaling pathway, Sphingolipid metabolism and Neurotrophin signaling pathway and cellular pathways, Lysosome, Adherens junction, Autophagy) (Fig. 3A and Table S2). Inflammatory pathways (e.g., amyloid-beta formation [GO:0034205]) also show enrichment although they are not among the top enriched GO terms and KEGG pathways (Table S2). Enriched TFs included zinc fingers (e.g., ZKSCAN8 and ZNF507) as well as MYRF, a myelin regulatory factor, and Bromodomain and PHD Finger Containing Protein 1, a transcriptional regulator implicated in neuron remodeling that has also been implicated in Alzheimer’s disease progression [53] (Table S3).

Cluster 2 contained 1176 genes (43.9% of DEGs) downregulated across age (Fig. 2C). GO analysis found enrichment for 136 pathways after FDR, including GO term enrichment for aerobic electron transport chain (GO:0019646), mitochondrial ATP synthesis coupled electron transport (GO:0042775), and mitochondrial respiratory chain complex I assembly (GO:0032981) and several neurobiological categories (Table S2). Enriched KEGG pathways for these genes listed several neuro-related diseases including Parkinson disease, Alzheimer disease, and pathways of neurodegeneration (Fig. 3B and Table S2). Enriched TFs included zinc fingers (e.g., DPF1, ZNF428, SCRT1, GTF3A, and ZNF511) as well as Peptidyl-prolyl cis-trans isomerase NIMA-interacting 1, an enzyme that has been implicated in the pathogenesis of Alzheimer’s disease and many cancers [54] (Table S3).

Cluster 3 contained 159 genes (5.9% of DEGs) with nonlinear trends in expression; specifically, these genes were found to increase in expression before decreasing later in life with an inflection point centered around ~ 10 years, as estimated from the LOESS-smoothed expression curve (9.5 years) (Fig. 2D). GO analysis did not identify any significantly enriched GO terms for the genes in this cluster after *p*-value correction and KEGG pathway enrichment identified only 1 significantly enriched pathway: amphetamine addiction (Table S2). The top enriched TF for the genes in cluster 3 was zinc finger ANKZF1 (Table S3). Indeed, zinc fingers were highly enriched for the genes in this cluster as several others appeared in the top 10 enriched TFs (e.g., ZNF692, RBCK1, ZNF540, CXXC1, GLI4, and ZBED5) (Table S3). In addition, Neuronal Differentiation 6, a basic helix-loop-helix transcription factor involved in the development and differentiation of the nervous system [55], was also enriched.

Cluster 4 contained 329 genes (12.3% of DEGs) which also demonstrated nonlinear trends in gene expression; specifically, these genes decreased in expression before increasing again later in life with a similar inflection point centered around ~ 10 years, as estimated from the LOESS-smoothed expression curve (9.2 years) (Fig. 2E)—exhibiting an inverse pattern to cluster 3. GO analysis showed enrichment for genes related to histone modifications (GO:0070932) and transcriptional regulation (GO:1903507) (Table S2). There were no KEGG terms significantly enriched for the genes in this cluster after *p*-value correction (Table S2). The top enriched TF for the genes in this cluster was Homeobox protein Nkx-6.2, a TF implicated in myelination regulation and associated with spastic ataxia in humans, a disease characterized by a general hypomyelination of the central nervous system which can lead to a number of neurological complications or death [56, 57] (Table S3). Other enriched TFs included zinc fingers (e.g., ZNF853 and ZBTB47), oligodendrocyte transcription factor OLIG1, MYRF, and box genes SOX10 and SOX8 which are implicated in brain development and function [58].

We also aimed to determine whether these identified expression patterns were maintained when looking within sex. To accomplish this, we divided our 88 individuals into males (*N* = 33) and females (*N* = 55) and reran our differential expression analyses (Fig. S5). With these models, we identified 2068 DEGs (~ 80% overlap with original approach) in females (Table S4 and Fig. S6) and 1742 DEGs (~ 65% overlap with original approach) in males (Table S5 and Fig. S7) (FDR < 0.05). Hierarchical clustering analysis suggested that differentially expressed genes for both females and males grouped into four broad expression patterns that largely mirrored the patterns of the original approach (see Supplemental Results and Discussion).

### Older adults exhibit increased variance in expression compared to younger adults

We identified 24 genes (FDR < 0.05) which exhibited greater variance in individuals aged ~ 10–20 years (*n* = 35) compared to 291 genes in individuals > 20 years of age (*n* = 29). While pathway analysis did not identify any significantly enriched GO terms in the first group of genes, we identified 39 significantly enriched GO terms for the 291 genes in the second group and a number of them are implicated in brain-related processes (e.g., transport across blood–brain barrier [GO:0150104] & positive regulation of neurogenesis [GO:0050769]) (Table S6). In addition, mean variance across genes was found to be significantly higher in individuals > 20 years of age (0.0645) compared to individuals aged 10–20 years (0.0528) (*t* =  − 32.89, df = 9550, *p* = 2.2e − 16, mean difference =  − 0.170). Collectively, these findings may suggest a disruption in gene regulatory control of neuronal processes associated with increased age.

### Deconvolution analysis identifies known age-related changes in oligodendrocytes and glutamatergic neurons

To infer how cell types change across age in the rhesus hippocampus, we employed cell-type deconvolution analysis using CIBERSORTx. This analysis revealed that the majority of our 12 reported cell types, which include neurons and glial cells (as denoted in the signature matrix based on the hippocampal dataset of [47]), do not appear to significantly change in proportion across age (Table S7).

**Fig. 4:**
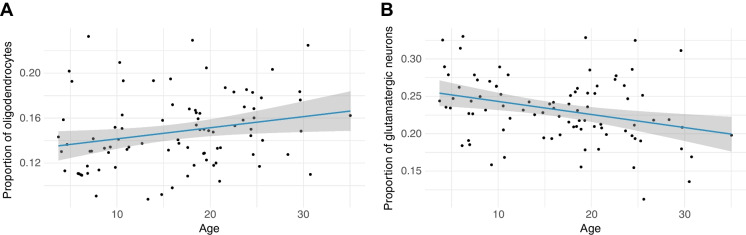
Proportion of significant cell type change across age. **A** Linear regression of oligodendrocyte (*β* = 0.00099, 95% CI [0.00012, 0.00186], *p* = 0.026) and **B** glutamatergic neuron (*β* =  − 0.0017, 95% CI [− 0.0029, − 0.0006], *p* = 0.003) cell type proportion change across age

However, two cell types appear to differ in abundance across the lifespan: oligodendrocytes significantly increase in proportion across age (*β* = 0.00099, 95% CI [0.00012, 0.00186], *p* = 0.026) while glutamatergic neurons significantly decrease in proportion across age (*β* =  − 0.0017, 95% CI [− 0.0029, − 0.0006], *p* = 0.003) (Fig. 4). The observed increase in oligodendrocytes is consistent with previous observations in rhesus macaques [59, 60] although it is not unique to the hippocampus. The observed decrease in glutamatergic neurons is consistent with decreases in glutamate concentration observed with age in human brains [61–63]. We did not observe a significant increase in microglia proportions associated across age as we might expect given increased activation and density of microglia with age in human brains [64], but see [60, 65, 66] and Supplemental Results & Discussion.

To examine gene expression changes independent of changes in cell-type proportions, we performed an additional ARIMA analysis controlling for the effects of estimated cell-type proportions. This analysis identified 2177 age-associated DEGs (FDR < 0.05). Hierarchical clustering analysis revealed that these genes largely fit four broad expression patterns (Fig. S8). The expression patterns largely mirrored the patterns of the original (non-cell-type corrected) approaches with the first two clusters exhibiting largely linear (i.e., monotonic) trends (cluster 1 = 1026 genes; cluster 2 = 845 genes) while the other two clusters exhibited nonlinear trends (cluster 3 = 188 genes; cluster 4 = 118 genes) (Fig. S9). Approximately ~ 71% of these genes overlapped with the genes identified by our original approach. Most of the overlap was found between the linear clusters (cluster 1 overlap = 97.1%; cluster 2 overlap = 86.9%) with no overlap identified in the nonlinear clusters (cluster 3 overlap = 0%; cluster 4 overlap = 0%). This approach also showed a moderate similarity in effect size to the original model (*r* = 0.32) as determined by calculating the correlation between the two sets of coefficients for the common genes, focusing on the diagonal elements to compute an average correlation value that summarizes the similarity between the two models. This may suggest that cross-sectional differences in cell type composition are impacting fluctuations in gene expression between time points, despite strong overlap in gene expression patterns at large—particularly in the linear clusters. However, this interpretation should be approached with caution, as it is based on the signature matrix used (see Supplemental Discussion).

## Discussion

We characterized age-associated gene expression changes in the hippocampus of rhesus macaques across the lifespan and identified a set of age-associated genes that exhibit nonlinear patterns of expression-findings that may provide novel insight into the molecular mechanisms associated with brain aging during midlife. Specifically, these nonlinear patterns revealed expression minima (or maxima) at ~ 10 years of age (approximately 30 years in humans) (Fig. 2), suggesting this as a key transition point between molecular development and decline of the hippocampus. We refer to this inflection point as ~ 10 years throughout the main text to provide a clear and interpretable age division, while acknowledging that it does not represent a precise biological transition common to all individuals. Importantly, this finding supports growing interest in characterizing more dynamic and nonlinear patterns of aging at the molecular level. For example, a recent multi-omics profiling study of human biological samples (e.g., blood, stool, skin swab, oral swab, and nasal swab samples) found that many molecular patterns of aging are nonlinear, with periods of significant change occurring during mid-life [35].

Studies have shown that midlife in humans is associated with a variety of biological and environmental factors that cumulatively contribute to the health of the brain during old age [67, 68]. The physiological changes occurring during this period (e.g., metabolic alterations), alongside lifestyle factors, can have a profound impact on the hippocampus, potentially predisposing individuals to cognitive decline and increasing their risk of neurodegenerative conditions such as Alzheimer’s disease [2, 8]. There are two non-mutually exclusive explanations for the shift in expression trajectories we observed in rhesus at ~ 10 years of age.

First, the signal reflects the completion of brain maturation in macaques. It is widely understood that human brains undergo a “rewiring” process and do not reach full maturation until the age of 25 [69, 70]. It is therefore possible that the molecular shift identified in our nonlinear expression trajectories is reflective of “completed” maturation of the rhesus macaque brain. This hypothesis is supported by our enrichment results. Specifically, our TF enrichment analysis found that DEGs that shift expression at ~ 10 years are associated with box proteins (SOX10 and SOX8), transcription factors important for neural crest development and brain differentiation generally. Additionally, [71] identified *ETNPPL* as a primate-specific neural stem marker playing an important role in adult hippocampal neurogenesis. We found this gene to be differentially expressed across age specifically exhibiting nonlinear patterns of expression (cluster 4). This further highlights a potential transition point between the developing and mature rhesus macaque hippocampus.

Second, the signal reflects changes in white matter volume associated with aging. Studies have revealed that white matter (i.e., regions containing myelinated axons) volume peaks at approximately 30 years of age in humans and declines thereafter [72–75], with the greatest degree of decline being detected in the prefrontal cortex and anterior corpus callosum [76–78]. Indeed, a decrease in white matter volume is a hallmark of general primate brain aging [77, 79]. In rhesus macaques, [80] identified that white matter volume peaks around ~ 10 years of age matching known patterns in humans. Additionally, [81] found that white matter volumes in macaques exhibit an arc-shaped trajectory with peaks occurring in middle age. These trends directly correspond to the shift observed in our nonlinear clusters and suggest that the genes in these clusters may be impacted by or drive this change in white matter volume. This may be the most likely explanation for the molecular shift observed in our nonlinear clusters as it is supported by the results of our cell-type deconvolution analysis. Specifically, the observed oligodendrocyte proportion increase associated with increased age. The primary function of oligodendrocytes in the brain is to produce and maintain myelin which ensheathes axons in order to increase speed and efficiency of axon signal conduction [82]. Increased oligodendrocyte proportion associated with age is argued to be a compensatory mechanism to combat the detrimental effects of white matter loss through remyelination [83]. TF enrichment analysis also supports this hypothesis with TFs implicated in myelin regulation (MYRF) and oligodendrocyte differentiation (OLIG1 and OLIG2) associated with DEGs exhibiting nonlinear patterns of expression (DEGs in clusters 3 and 4).

Definitions of midlife vary with some studies specifying the midlife period as between the ages of 40 and 65 years in humans [84] and others more broadly referring to midlife as a period between youth and old age [85]. There is even less clarity when defining this period across species. For example, many descriptions of midlife in macaques simply reference adulthood or middle of the lifespan (e.g., 86). Rhesus macaques age at roughly 2–3 times the rate of humans and with sexual maturity typically occurring between 2.5 and 4.5 years, full body size being reached between 5 and 7 years, and a median lifespan of ~ 27 years [26, 87, 88]. For the sake of this study, we broadly define midlife in rhesus macaques as referring to any age between sexual maturation (~ 3–6 years) and old age (~ 25 years) [88, 89]. Our study suggests that this general timepoint (and the comparable timepoint in humans) could be an important transitionary period between normal hippocampal development and age-associated decline and thus may provide insight into the mechanisms that underpin the susceptibility and resilience of the hippocampus to age-related pathologies. Indeed, midlife represents a pivotal period in the human lifespan with regard to health outcomes, serving as a critical juncture that can significantly influence the aging trajectory of the hippocampus [90].

Similar to others, we found that the majority of differentially expressed genes exhibited linear trends in expression and are enriched for GO terms and pathways known to be upregulated (e.g., transcriptional regulation and inflammatory pathways; Table S2) or downregulated (e.g., metabolism and mitochondrial dysfunction; Table S2) with age respectively. Specifically, previous research has shown that inflammatory/immune response genes and pathways are upregulated in the hippocampus of older rodents [91, 92], rhesus macaques [93], and humans [94–96]. This pattern may relate to increased microglial activation and dysfunction during aging [97, 98]. Importantly, exaggerated pro-inflammatory responses associated with age in humans [99] can increase susceptibility to neurodegenerative diseases, as chronic brain inflammation is a pathological hallmark of Alzheimer’s disease [100]. Previous studies have also shown that genes involved in synaptic function, neuronal signaling, and synaptic plasticity are downregulated in the hippocampus of older primates [101]. In addition, genes involved in neural development, signaling pathways, and metabolic processes, which indicate a shift in cellular metabolism and function with age, have also been shown to be downregulated with age in the hippocampus of rodents [102].

Interestingly, KEGG pathways for neuro-related diseases (e.g., Parkinson disease & Alzheimer’s disease) were also enriched in DEGs downregulated across age (Table S2), identifying a set of genes that may be particularly relevant to understanding resilience mechanisms in aging primates. Many of these genes are involved in mitochondrial function, including regulators of apoptosis (e.g., *BAX*, *HTRA2*, *TOMM40*, *MCU*, *HSD17B10*) and components of oxidative phosphorylation (e.g., *ATP5F1C*, *ATP5F1E*, *COX7B*, *COX8A*), which are critical for maintaining cellular energy balance and neuronal health. Although mitochondrial dysfunction is a hallmark of aging and a key feature of neurodegenerative disease in humans [103], the downregulation of these genes in aging macaques may reflect an alternative regulatory trajectory that reduces apoptotic and metabolic stress to support neuronal resilience.

While this study was not designed to test the functional consequences of age-related gene expression changes directly, our findings highlight several candidates that warrant future investigation through functional validation approaches. For example, *BAX* is a pro-apoptotic member of the BCL2 family that promotes mitochondrial outer membrane permeabilization and initiates caspase activation during apoptosis [104, 105]. In humans, studies show that *BAX* expression is significantly elevated in the frontal cortices of individuals with Alzheimer’s disease compared to those with normal aging, suggesting that increased *BAX* expression contributes to neuronal vulnerability [106]. In contrast, downregulation of *BAX* in the aging macaque hippocampus, albeit a different brain region, may reflect an adaptive shift toward lower apoptotic sensitivity, preserving neuronal viability with age. Thus, *BAX* downregulation in macaques could represent a brain-specific resilience mechanism, consistent with prior findings that macaques maintain higher expression of genes related to neurogenesis and synaptic plasticity into adulthood [107], and do not exhibit the widespread neuronal loss characteristic of Alzheimer’s disease [108]. Understanding the role these genes (Table S2) play in hippocampal aging and how they are differentially regulated across species may reveal important clues to human-specific vulnerability to neurodegeneration and disease [101].

The age-associated increase in gene expression variance was also consistent with previous studies which observed this phenomenon both in the brain and in tissues throughout the body both in humans [109] and rhesus macaques [60]. Age-related increases in variance of expression are thought to be explained by decline in gene regulatory control mechanisms (i.e., dysregulation) which contribute both to general aging and age-associated diseases [110, 111]. We found that genes which exhibited increased variance in expression for individuals > 20 years of age were enriched for brain-related GO terms including transport across blood-brain barrier, positive regulation of neurogenesis, and synapse pruning (Table S6). These terms provide potential biological mechanisms which may be impacted by or implicated in the loss of gene regulatory control associated with age. Indeed, the hippocampus is distinct from other brain regions in that it is one of the first to suffer breakdown in the blood–brain barrier [19]. Our results suggest that increased variance in expression may relate to this breakdown, although the mechanism and direction of interaction require further investigation.

Finally, several genes known to be integral in facilitating the anti-aging effects of calorie restriction also demonstrated age-associated patterns of differential expression in the hippocampus of rhesus macaques. Many of these transcripts follow linear trajectories in expression (e.g., *IGF1R* & *FOXO3* are upregulated across age); however, two exhibit nonlinear trajectories of expression. Specifically, *SIRT1* and *SIRT2* have more “U-shaped” expression trajectories, reaching their lowest expression levels at ~ 10 years of age (Fig. S10). The sirtuin genes are argued to play pivotal roles in neuroprotection and cellular senescence [112]. Both *SIRT1* and *SIRT2* encode NAD + -dependent protein deacetylases involved in the regulation of glucose metabolism, insulin signaling, and oxidative stress protection [113, 114]. Sirtuin 1 promotes neuronal health during aging through supporting axonal elongation, neurite outgrowth, dendritic branching, and memory formation by modulating synaptic plasticity [115]. Sirtuin 2 deacetylates the transcription factor Forkhead Box Protein O3 in response to oxidative stress and calorie restriction, promoting cellular survival pathways [108]. Given their importance in anti-aging/neuroprotection [116], midlife changes in these genes and pathways warrant further study.

This study provides valuable insights into molecular changes occurring during midlife in the aging rhesus macaque hippocampus; however, several limitations of the dataset warrant consideration. First, the analysis was conducted on RNA derived from previously banked whole tissue samples. This approach provides a broad molecular overview but is inherently limited in its resolution. While we include deconvolution analyses, these types of approaches are only able to infer cellular changes and do not allow for direct observation of cell-specific changes and insights that would be possible using single-cell RNA sequencing. Second, the absence of correlative behavioral data limits the ability to control for cognitive or functional variability that might otherwise provide additional context for the observed molecular changes. Third, the samples were collected over a 25-year period, introducing potential variability. Nonetheless, all samples included in the study were confirmed to be free of overt neuropathologies, and the post-mortem interval prior to tissue storage was minimized to ensure sample integrity. Despite these limitations, this study addresses a critical gap in understanding the molecular landscape of hippocampal aging in primates, a topic largely uncharacterized due to the scarcity of available samples. Moreover, while the study is not longitudinal, the inclusion of a large sample size with replicates of individuals at similar ages strengthens the reliability of the findings.

This study characterizes transcriptomic aging in the hippocampus of rhesus macaques, revealing critical molecular transitions that occur during midlife. We identified both linear and nonlinear patterns of gene expression that provide novel insights into the aging process, particularly during this pivotal stage of life. The significant shifts in gene expression observed in rhesus macaques at ~ 10 years of age (~ 30 years of age in humans) suggest that midlife may represent a critical juncture in hippocampal molecular aging. These nonlinear trajectories, coupled with the observed changes in oligodendrocyte proportions and white matter volume, highlight the potential role of cellular composition in influencing gene expression during this period. Furthermore, our results align with broader patterns observed in humans and other primates, underscoring the importance of midlife as a period where molecular and cellular changes may predispose the brain to age-related pathologies [103]. Importantly, this study emphasizes the need for future research to dissect the specific roles these differentially expressed genes play in shaping aging phenotypes and age-associated diseases, especially as nonlinear patterns of gene expression may uncover novel mechanisms driving susceptibility or resilience to neurodegenerative conditions.

## Supplementary Information

Below is the link to the electronic supplementary material.

Supplementary file1 (DOCX 7033 KB)

## Data Availability

Raw RNAseq data for this project is publicly available (Accession: PRJNA1164722). All code used in the analysis is available at https://github.com/tannerndrsn4/Hippocampus-RNAseq-Nonlinear-Modeling/tree/main.
