# Supplementary material for: Patterns of transcriptomic aging in the hippocampus of rhesus macaques highlight midlife transitions
Source: GeroScience. 2025 Aug 18;48(2):2913–29. doi: 10.1007/s11357-025-01834-z (PMC12972383; doi:10.1007/s11357-025-01834-z)
Supplement: Supplementary file 1 — Supplementary file1 (DOCX 7033 KB) [file 11357_2025_1834_MOESM1_ESM.docx]

**Title:** Patterns of transcriptomic aging in the hippocampus of rhesus macaques highlight midlife transitions

**Journal:** GeroScience

**Author List:** Tanner J. Anderson^1^, Marina M. Watowich^2,3^, Kenneth L. Chiou^4,5,6^, Elisabeth A. Goldman^7^, Sam Peterson^8^, Jordan A. Anderson^9^, Noah Snyder-Mackler^4,5,10,11^, Lucia Carbone^12,13,14^, Steven G. Kohama^8^, and Kirstin N. Sterner^1*^.

**Affiliations:**

1. Department of Anthropology, University of Oregon

2. Department of Biological Sciences, Vanderbilt University

3. Department of Biology, University of Washington

4. Center for Evolution and Medicine, Arizona State University

5. School of Life Sciences, Arizona State University

6. Department of Biology, University of Alabama at Birmingham

7. Cancer Early Detection Advanced Research Center, Knight Cancer Institute, Oregon Health & Science University

8. Division of Neuroscience, Oregon National Primate Research Center

9. Institute of Ecology and Evolution, University of Oregon

10. School of Human Evolution and Social Change, Arizona State University

11. Neurodegenerative Disease Research Center, Arizona State University

12. Department of Medicine, KCVI, Oregon Health & Science University

13. Department of Molecular and Medical Genetics, Oregon Health & Science University

14. Division of Genetics, Oregon National Primate Research Center

*****Corresponding author ([ksterner@uoregon.edu](mailto:ksterner@uoregon.edu))

**SUPPLEMENTAL TABLE OF CONTENTS**

1. SUPPLEMENTAL METHODS -- (3)
2. SUPPLEMENTAL RESULTS – (5)
3. SUPPLEMENTAL DISCUSSION – (6)
4. SUPPLEMENTAL TABLES – (8)
   1. Supplemental Table Descriptions – (8)
5. SUPPLEMENTAL FIGURES – (9)
   1. Figure S1 – (9)
   2. Figure S2 – (10)
   3. Figure S3 – (11)
   4. Figure S4 – (12)
   5. Figure S5 – (13)
   6. Figure S6 – (14)
   7. Figure S7 – (15)
   8. Figure S8 – (16)
   9. Figure S9 – (17)
   10. Figure S10 – (18)

**Supplemental Methods**

Preliminary data exploration

We performed preliminary data exploration using PCA analysis. The analysis highlighted a potential batch effect in the data along with the presence of outliers (**Fig. S1**).

To identify and address the potential technical effects in the data, we took the following steps. First, we tested whether there was presence of choroid plexus tissue contamination. The choroid plexus is a brain tissue responsible for making cerebrospinal fluid in the lateral and fourth ventricles of the brain (in close proximity to the hippocampus) and, due to its complex structure, it can be a source of contamination during routine dissections [1]. Olney et al. (2022) identified the genes *TTR*, *FOLR1* and *PRLR* as being markers of choroid plexus contamination. An expression plot for these genes identified a subset (n=6) of individuals which exhibited higher than expected expression levels (**Fig. S2**).

Next, we performed hierarchical sample clustering analysis using a distance matrix to identify whether any individuals clustered distinctly from the rest of the dataset. The clustering analysis identified 2 individuals clustering distinctly from the remainder of the dataset (**Fig. S3**).

Due to evidence of choroid plexus tissue contamination and high degree of outlier status, we chose to remove 8 individuals (4 female & 4 male) from the dataset and all subsequent analysis (**Table S1**).

Next, we performed variance partitioning analysis using the R package variancePartition [2]. This provides another method of assessing the effects of metadata variables on explaining the variance in the dataset. Our analysis identified the chip denotation – an identifier held over from sequencing – in our metadata as being the greatest explainer of the variance in the dataset compared to other metadata variables sex and age (**Fig. S4**). Therefore, we regressed out the effect of chip in our dataset and performed all subsequent analyses on the residuals.

To further assess potential residual technical confounding, we tested associations between the first 10 principal components (PCs) — representing the ~68% of cumulative variation in gene expression — and available technical variables, including RNA Integrity Number (RIN), Bioanalyzer Nano concentration, and RNA remaining after quality assessment. These analyses were performed using linear models on the subset of samples with available metadata (e.g., RIN data were available for 88 individuals, not all of whom were included in the differential expression analysis). After applying false discovery rate (FDR) correction for multiple testing, no significant associations were detected (FDR < 0.05), and these technical variables were not included as covariates in the differential expression models.

Differential expression analysis

To identify age-associated trajectories in hippocampal gene expression, we employed an ARIMA (Autoregressive Integrated Moving Average) modeling pipeline adapted from Márquez et al. (2020). ARIMA models are widely used in time series analysis for their ability to capture both autoregressive patterns and moving average effects, while accounting for non-stationarity through differencing. In the context of gene expression, this approach enables detection of dynamic, potentially nonlinear trends across chronological age [3].

Normalized and residualized RNA-seq expression values (with effects of sex and technical covariates regressed out) were first ordered by age. When multiple samples shared the same age, their expression values were averaged to ensure a single measurement per time point. These age-aligned gene expression series were then converted into regular time series objects using the zoo and ts frameworks in R. Each gene’s time series was independently modeled using the auto.arima function from the forecast R package, which selects the best-fitting ARIMA configuration (p,d,q) based on the lowest Akaike Information Criterion (AIC) value. Optional Box-Cox transformations were applied to stabilize variance where appropriate. We retained models only if they met two quality control criteria: (1) inclusion of at least one autoregressive or moving average term (i.e., evidence of temporal structure), and (2) residuals that passed the Ljung-Box test for independence (FDR > 0.05), indicating good model fit without autocorrelated error. Genes that passed both filters were considered to exhibit non-random, age-associated trends in expression. To facilitate interpretation and comparison across ages, we applied LOESS (locally estimated scatterplot smoothing) interpolation to the fitted ARIMA values, generating smooth trajectories over a continuous age range. These smoothed predictions were used in downstream analyses of age-related expression patterns. This modeling approach is advantageous because it does not assume linearity or a fixed trend structure a priori, and it flexibly accommodates temporal complexity in gene regulation across the aging process.

**Supplemental Results**

Identification of DEGs in female dataset and male dataset

We aimed to assess whether the same molecular signals identified were held up when looking within sex. To address this, we divided our 88 individuals into males (N = 33) and females (N = 55) and reran our analyses. With these models, we identified 2,068 differentially expressed genes in females and 1,742 genes in males (FDR < 0.05). Hierarchical clustering analysis suggested that the genes for both females (**Fig. S5A**) and males (**Fig. S5B**) grouped into 4 broad expression patterns.

Description of DEGs identified in the female dataset

Beginning with the 2,068 differentially expressed genes identified in the female dataset, the 4 broad expression patterns identified largely reflected what was reported in our previous ARIMA approach. Indeed ~80% of these genes overlapped with the genes identified in the previous approach. The first 2 clusters identified genes exhibiting linear trends in expression (**Fig. S6; A and B**) while the other 2 identified genes exhibiting nonlinear trends in expression (**Fig. S6; C and D**). Additionally, while the nonlinear clusters identified a shift in expression around ~10 years of age, they also identified shifts at ~19 and ~25 years (**Fig. S6; C and D**).

Gene set and TF enrichment analyses for the genes in the linear clusters largely reflected the results reported in the original approach. For the nonlinear clusters, we were also able to identify significantly enriched GO terms after p-value correction. Specifically, for Female Cluster 3, the top 3 enriched GO terms were lipid modification (GO:0030258), mRNA splice site selection (GO:0006376) and positive regulation of establishment of protein localization to telomere (GO:1904851). TF enrichment contained additional enrichment for *DACH2*, a TF involved in cell fate determination and linked to premature ovarian failure [4] (**Table S3**). For Female Cluster 4, the top 3 enriched GO terms were myelination (GO:0042552), positive regulation of transcription by RNA polymerase II (GO:0045944) and positive regulation of transcription, DNA-templated (GO:0045893) (**Table S6**). TF enrichment results largely mirrored those reported in the original ARIMA approach (**Table S3**).

Description of DEGs identified in the male dataset

For the 1,742 differentially expressed genes identified in the male dataset, the 4 broad expression patterns identified also reflected what was reported in the original ARIMA approach. Approximately ~65% of these genes overlapped with the genes identified in the original approach with the first 2 clusters identified exhibiting monotonic (i.e., linear) trends in expression while the other 2 exhibited nonlinear trends in expression. The nonlinear clusters also identified a shift in expression around ~10 years similar to our original approach (**Fig. S7**).

Gene set enrichment analysis provided slightly differing results compared to the previous approaches. For Male Cluster 1, the top 3 significantly enriched GO terms were identified as bleb assembly (GO:0032060), neutrophil degranulation (GO:0043312) and neutrophil activation involved in immune response (GO:0002283). For Male Cluster 2, the top 3 significantly enriched GO terms were regulation of cation channel activity (GO:2001257), regulation of neurotransmitter receptor activity (GO:0099601) and axonogenesis (GO:0007409). There were no significantly enriched GO terms identified for Male Cluster 3. Lastly, the top 3 significantly enriched GO terms for Male Cluster 4 were protein transport (GO:0015031), response to insulin (GO:0032868) and cellular response to insulin stimulus (GO:0032869) (**Table S7**). TF enrichment results for the genes in the male clusters largely upheld the patterns reported in the original and female specific ARIMA approach (**Table S3**).

**Supplemental Discussion**

Sex-specific trends in gene expression

The DEGs identified in the female- and male-specific ARIMA approaches largely uphold the patterns identified in the original ARIMA approach. This is consistent with observed patterns of sex-biased gene expression in the macaque brain. Specifically, DeCasien et al. (2022) found that sex-biased differences in gene expression were more prevalent between brain regions rather than within regions. Therefore, we would not expect to see major differences between females and males in terms of the genes showing age-associated patterns of differential expression. Where we do observe differences is in how these genes are being used. Despite strong conservation, the timing and direction of expression differs between the sexes and highlights additional time points which may be relevant in shaping hippocampal aging within sex.

When examining the nonlinear clusters for both females and males, both identify a shift in expression centered around ~10 years of age, consistent with the combined dataset. The female nonlinear clusters also identified shifts in expression centered around the ages of ~19 and ~25 years (**Fig. S6**). Shifts in expression at ~19 and ~25 years of age could be explained by female individuals entering perimenopause and menopause respectively. Perimenopause and menopause have been demonstrated to significantly impact cognition and brain health in both humans and rhesus macaques [6–9]. Specifically, perimenopause is characterized by disruptions in oestrogen production –a hormone important in glucose uptake and energy production [9]. These disruptions contribute to a period of metabolic dysfunction as the brain must adjust to existence without oestrogen and indeed these perimenopausal changes are argued to be linked to Alzheimer’s disease risk [7,10]. Indeed, our TF enrichment results lend support to this explanation. Specifically, binding motifs for DACH2, a TF involved in cell fate determination and linked to premature ovarian failure [4] were enriched among genes exhibiting nonlinear patterns of expression in the female-specific dataset.

While relevant hormone data and menses status for the specific rhesus macaque females included in this study were not available, an adjacent study examining changes in female reproductive hormones with age within rhesus macaque females housed at the ONPRC specifically found that, in general, female macaques showed normal to irregular or elongated cycles in their early to mid 20s while after 25 years they became intermittent [11]. As a result, the observed molecular shifts in older females could relate to changes in reproductive hormones/menses status associated with age.

An alternative explanation for the shift observed around ~25 years of age in females could have to do with the specific life history of rhesus macaques. The WNPRC classifies rhesus macaques over the age of 25 years as being post geriatric and “old age” which is reflected by a number of physiological changes including in the brain [12]. We did not identify any additional shifts in expression in the male nonlinear clusters, however the lack of a signal in males does not preclude its existence. Our dataset is female biased and gets more female-biased with age (**Fig. 1A**). Indeed, there is only 1 male in our dataset >25 years of age. Therefore, we might observe a similar signal if older males were sampled.

Cell-type deconvolution results: impact and limitations

We did not observe a significant increase in microglia proportions associated across age as we might expect given increased activation and density of microglia with age in human brains [13]. Additionally, microglia are a known cell-type demonstrated to be affected by the aging process at the gene expression level in the primate hippocampus [14]. However, this may not be entirely surprising given a handful of studies that indicate microglial activation and density differs in nonhuman primates compared to humans. Specifically, studies concerning marmosets [15], rhesus macaques [16–18] and chimpanzees [19] show no significant change with age. Indeed, our results are consistent with these reports and appear to support the argument of a species-specific difference in microglia trajectory for humans. This argument is challenged however by conflicting reports which suggest older rhesus macaques (≥20 years) share a similar aging pattern to humans in terms of microglial densities [20,21]. These inconsistencies highlight the need for further research in establishing normal age-related microglial changes amongst primates. Microglia serve as the resident immune cells of the brain and their increased activation contributes to increased inflammatory responses [22]. With chronic inflammation being a hallmark of several neurodegenerative diseases (e.g., Alzheimer’s disease) [23], establishing microglial trajectories in normal nonhuman primate brain aging may be necessary in investigating the human-specific vulnerability to these diseases.

An alternative explanation for the lack of change in overall microglia proportions could be that microglia activation associated with age may be driven by relative proportions of particular microglia subtypes [24]. Meaning, while there may not be significant changes in microglia proportions across age overall, certain subtypes relevant in brain aging/health might be. Of particular interest would be disease-associated microglia. Future studies should attempt to characterize microglia subtype changes with age to assess their role in shaping rhesus macaque hippocampal aging.

When performing our differential expression analysis while controlling for cell-type proportions, our results largely upheld the patterns identified in our original ARIMA approach (**Fig. S8 & S9)**. This suggests that age-related patterns in differential gene expression cannot be completely explained by differences in cell-type proportions. Notably, the expression trajectories for all 4 identified clusters appear to exhibit less interindividual variation than the expression trajectories identified in the original approach (controlling for sex). This might suggest that fluctuations (i.e., seemingly dynamic changes between neighboring age points) may be explained by individual differences in cell type proportions rather than distinct cell-type changes with age. This in turn offers a more pointed view at age-related gene expression trajectories independent of interindividual variation in terms of cell-type proportions.

While revealing, cell-type deconvolution analyses have important limitations. Our approach infers cell-type, and our findings should be tested using more direct approaches (e.g., single-cell RNAseq). The estimated cell-type proportions and their changes over the lifespan are influenced by marker genes that themselves may be differentially expressed within cell types across the lifespan, thereby confounding our estimation of relative proportions. Additionally, to avoid introducing further error in cell-type predictions, we opted to focus here on predicting cell types at a broad level of cell class, precluding our ability to examine proportional changes in more granular, but potentially important cell types and subtypes. Therefore, we view the results, and their potential implications discussed here, as hypotheses for future research to investigate.

**Supplemental Tables**

*Supplemental table descriptions*

**Table S1.** Metadata for all 96 rhesus macaque hippocampus samples. Green bars highlight eight individuals removed from the dataset and subsequent analysis due to choroid plexus contamination and outlier status.

**Table S2.** enrichR gene ontology results for all 2,679 genes identified by the ARIMA modeling approach controlling for sex. Includes enriched GO biological processes (BP) and KEGG pathways. Ontology results are separated by cluster.

**Table S3.** Transcription factor motif enrichment results using ChIP-X Enrichment Analysis 3 (ChEA3). Includes results for full dataset (controlling for sex), female individuals and male individuals respectively. Enrichment results are separated by expression cluster.

**Table S4.** enrichR gene ontology results for all 2,608 genes identified by the ARIMA modeling approach using just female individuals. Includes enriched GO biological processes (BP) and KEGG pathways. Ontology results are separated by cluster.

**Table S5.** enrichR gene ontology results for all 1,742 genes identified by the ARIMA modeling approach using just male individuals. Includes enriched GO biological processes (BP) and KEGG pathways. Ontology results are separated by cluster.

**Table S6.** Significantly enriched GO terms for 291 genes exhibiting greater variance in expression in older individuals (>20 years).

**Table S7.** CIBERSORTx cell-type deconvolution results for all individuals and genes in the dataset.

**Supplemental Figures**

**Fig. S1** Sample distribution and PCA analysis of 96 hippocampus samples. A) Distribution of ages for 96 hippocampus samples denoted by sex. B) PCA analysis of 96 hippocampus samples. Individuals denoted by sex, C) Individuals denoted by age (PC1 = 30.28%; PC2 = 15.07%)

**Fig. S2** Normalized expression plots of three known choroid plexus marker genes

**Fig. S3** Hierarchical sample clustering analysis using a distance matrix to detect outliers. Individual IDs 23207 and 20132 were the most separated by the analysis

**Fig. S4** Variance partitioning analysis of gene expression data (formula = gene expression ~ age + sex + chip)

**Fig. S5** Hierarchical clustering analysis shows 4 broad expression patterns for female and male DEGs. A) Cluster Dendrogram of all significantly differentially expressed genes as identified by ARIMA modeling for females and B) males respectively. Clusters utilized in analysis are denoted by red nodes

**Fig. S6** Expression trajectories of female gene clusters. A) Averaged expression trajectory for all genes (n=757) in Cluster 1 (upregulated across age), B) all genes (n=593) in Cluster 2 (downregulated across age), C) all genes (n=400) in Cluster 3 (first nonlinear age-associated cluster) D) and for all genes (n=318) in Cluster 4 (second nonlinear age-associated cluster)

**Fig. S7** Expression trajectories of male gene clusters. A) Averaged expression trajectory for all genes (n=698) in Cluster 1 (upregulated across age), B) all genes (n=861) in Cluster 2 (downregulated across age), C) all genes (n=31) in Cluster 3 (nonlinear age-associated cluster) D) and for all genes (n=152) in Cluster 4 (age-associated cluster)

**Fig. S8** Cluster Dendrogram of all significantly differentially expressed genes as identified by ARIMA modeling when controlling for cell-type proportion changes. Clusters utilized in analysis were denoted by red nodes

**Fig. S9** Averaged expression trajectories for genes in each distinct cluster when controlling for cell-type proportion changes. A) Cluster 1 contains 1,026 genes largely upregulated across age. B) Cluster 2 contains 845 genes largely downregulated across age. C & D) Clusters 3 and 4 contained 188 and 118 genes respectively and both exhibit nonlinear trends in expression

**Fig. S10** Fitted expression trajectories over the lifespan for A) *SIRT1* and B) *SIRT2* respectively. Expression results are from full dataset when controlling for sex

**Supplemental References**

1. Olney KC, Todd KT, Pallegar PN, Jensen TD, Cadiz MP, Gibson KA, Barnett JH, de Ávila C, Bouchal SM, Rabichow BE, Ding Z, Wojtas AM, Wilson MA, et al. Widespread choroid plexus contamination in sampling and profiling of brain tissue. Mol Psychiatry. Nature Publishing Group; 2022; 27: 1839–47.

2. Hoffman GE, Schadt EE. variancePartition: interpreting drivers of variation in complex gene expression studies. BMC Bioinformatics. 2016; 17: 483.

3. Brockwell, P. J., Davis, R. A. & Calder, M. V. Introduction to Time Series and Forecasting. Springer, 2002.

4. Bilgin EM, Kovanci E. Genetics of premature ovarian failure. Curr Opin Obstet Gynecol. 2015; 27: 167–74.

5. DeCasien AR, Chiou KL, Testard C, Mercer A, Valle JEN-D, Surratt SEB, González O, Stock MK, Ruiz-Lambides AV, Martinez MI, Unit CBR, Antón SC, Walker CS, et al. Evolutionary and biomedical implications of sex differences in the primate brain transcriptome. bioRxiv; 2022. p. 2022.10.03.510711.

6. Greendale GA, Derby CA, Maki PM. Perimenopause and Cognition. Obstet Gynecol Clin North Am. 2011; 38: 519–35.

7. Brinton RD, Yao J, Yin F, Mack WJ, Cadenas E. Perimenopause as a neurological transition state. Nat Rev Endocrinol. 2015; 11: 393–405.

8. Sorwell KG, Renner L, Weiss AR, Neuringer M, Kohama SG, Urbanski HF. Cognition in aged rhesus monkeys: effect of DHEA and correlation with steroidogenic gene expression. Genes Brain Behav. 2017; 16: 361–8.

9. Ledford H. How menopause reshapes the brain. Nature. 2023; 617: 25–7.

10. Wilson RS, Leurgans SE, Boyle PA, Bennett DA. Cognitive decline in prodromal Alzheimer disease and mild cognitive impairment. Arch Neurol. 2011; 68: 351–6.

11. Downs JL, Urbanski HF. Neuroendocrine Changes in the Aging Reproductive Axis of Female Rhesus Macaques (Macaca mulatta)1. Biology of Reproduction. 2006; 75: 539–46.

12. Simmons HA. Age-Associated Pathology in Rhesus Macaques (Macaca mulatta). Vet Pathol. 2016; 53: 399–416.

13. Edler MK, Mhatre-Winters I, Richardson JR. Microglia in Aging and Alzheimer’s Disease: A Comparative Species Review. Cells. 2021; 10: 1138.

14. Zhang H, Li J, Ren J, Sun S, Ma S, Zhang W, Yu Y, Cai Y, Yan K, Li W, Hu B, Chan P, Zhao G-G, et al. Single-nucleus transcriptomic landscape of primate hippocampal aging. Protein & Cell. 2021; 12: 695–716.

15. Rodriguez-Callejas JD, Fuchs E, Perez-Cruz C. Evidence of Tau Hyperphosphorylation and Dystrophic Microglia in the Common Marmoset. Front Aging Neurosci. 2016; 8: 315.

16. Peters A, Verderosa A, Sethares C. The neuroglial population in the primary visual cortex of the aging rhesus monkey. Glia. 2008; 56: 1151–61.

17. Kanaan NM, Kordower JH, Collier TJ. Age-related changes in glial cells of dopamine midbrain subregions in rhesus monkeys. Neurobiol Aging. 2010; 31: 937–52.

18. Chiou KL, DeCasien AR, Rees KP, Testard C, Spurrell CH, Gogate AA, Pliner HA, Tremblay S, Mercer A, Whalen CJ, Valle JEN-D, Janiak MC, Surratt SEB, et al. Multiregion transcriptomic profiling of the primate brain reveals signatures of aging and the social environment. Nat Neurosci. 2022; 25: 1714–23.

19. Edler MK, Sherwood CC, Meindl RS, Hopkins WD, Ely JJ, Erwin JM, Mufson EJ, Hof PR, Raghanti MA. Aged chimpanzees exhibit pathologic hallmarks of Alzheimer’s disease. Neurobiol Aging. 2017; 59: 107–20.

20. Robillard KN, Lee KM, Chiu KB, MacLean AG. Glial Cell Morphological and Density Changes Through the Lifespan of Rhesus Macaques. Brain Behav Immun. 2016; 55: 60–9.

21. Shobin E, Bowley MP, Estrada LI, Heyworth NC, Orczykowski ME, Eldridge SA, Calderazzo SM, Mortazavi F, Moore TL, Rosene DL. Microglia activation and phagocytosis: relationship with aging and cognitive impairment in the rhesus monkey. GeroScience. 2017; 39: 199–220.

22. Colonna M, Butovsky O. Microglia Function in the Central Nervous System During Health and Neurodegeneration. Annu Rev Immunol. 2017; 35: 441–68.

23. Balin BJ, Hudson AP. Etiology and pathogenesis of late-onset Alzheimer’s disease. Current allergy and asthma reports. Springer; 2014; 14: 417.

24. Paolicelli RC, Sierra A, Stevens B, Tremblay M-E, Aguzzi A, Ajami B, Amit I, Audinat E, Bechmann I, Bennett M, Bennett F, Bessis A, Biber K, et al. Microglia states and nomenclature: A field at its crossroads. Neuron. 2022; 110: 3458–83.
